# Assessment of serum proprotein convertase subtilisin/kexin type 9 in pediatric sepsis syndrome

**DOI:** 10.1038/s41598-024-65609-w

**Published:** 2024-07-07

**Authors:** Suzan Omar Mousa, Mohamed Farouk Afifi, Noha Anwar Hassuna, Michael Fekry Yassa, Hend Mohamed Moness

**Affiliations:** 1 Pediatric Department, Minia University Children Hospital, Faculty of Medicine, Minia University, El-Minya, 61111 Egypt; 2 Medical Microbiology and Immunology Department, Faculty of Medicine, Minia University, El-Minya, Egypt; 3 Clinical Pathology Department, Faculty of Medicine, Minia University Hospitals, Minia University, El-Minya, Egypt

**Keywords:** Innate immune response, PCSK9, LDL-cholesterol, Pediatric sepsis, Immunology, Medical research

## Abstract

Sepsis is a life-threatening condition that arises when the body's response to infection causes injury to its tissues and organs. Proprotein convertase subtilisin/kexin type 9 (PCSK9) is an enzyme released in response to the drop in cholesterol level occurring in sepsis. Our study aimed to evaluate the prognostic role of serum Proprotein convertase subtilisin/kexin type 9 (PCSK9) level in children with sepsis and severe sepsis. Sixty children were included in this study. They were divided into two groups: 30 children in the sepsis group and 30 in the severe sepsis group. Another 30 apparently healthy children were included as a control group. Blood samples were withdrawn from all included children for complete blood count (CBC), renal function tests (RFT), liver function tests (LFT), LDL-cholesterol (LDL-C), blood culture, and serum PCSK9. In this study, PCSK9 and LDL-C were higher in the two sepsis groups than in the control group (p < 0.05). They were also higher in the severe sepsis group than the sepsis group and in the non-survivors than in the survivors (p < 0.05). PCSK9 was positively correlated with length of hospital stay in surviving children (r = 0.67, p = 0.001) and had predicted significant hematological dysfunction (adjusted B =  − 96.95, p = 0.03). In conclusion, the PCSK9 assay can be used as a biomarker for bad prognosis in children suffering from clinical sepsis.

## Introduction

Sepsis, according to the "Third International Consensus Definitions for Sepsis and Septic Shock," is a systemic life-threatening syndrome characterized by the development of multi-organ dysfunction in the context of systemic infection^1^. Despite the substantial progress in recent years, sepsis is still a severe disease with difficult treatments for its manifestations and high mortality rates^2^.

Many studies suggested that the dysregulated host response to infection is the cause of the cascade of pathological sepsis-related events rather than only infection and pathogen overgrowth^1^.

Changes in cholesterol levels are considered a form of host-dysregulated response during inflammation and infection, which have been frequently reported and may also have some prognostic impact on sepsis^3^. Cholesterol and pathogen lipids were found to play an essential role in generating intracellular signals and, consequently, in the regulation of the systemic inflammatory response to different septic agents^4^. Low HDL-cholesterol levels have been associated with dysregulated inflammation and endothelial injury, which can result in the clinical manifestations of organ damage and poor sepsis outcomes^5–7^. In addition, LDL-cholesterol (LDL-C) has been shown to facilitate bacterial toxin clearance in sepsis^8^. As Lipoproteins can neutralize Pathogen-associated lipids in the blood, thereby reducing the pro-inflammatory response of immune cells. This 'sponge-like' innate immune function is present in very low-density lipoprotein (VLDL), low-density lipoprotein (LDL), lipoprotein(a), and high-density lipoprotein (HDL). Predominantly, HDLs bind Pathogen-associated lipids with the most excellent affinity. The mechanism of lipoprotein-lipopolysaccharide interactions still needs to be defined. Ultrastructural analyses show that the lipid component of lipoproteins is critical for sequestering the toxic lipid A moiety of lipopolysaccharide within phospholipid membranes of pathogens^9^.

Proprotein convertase subtilisin/kexin type 9 (PCSK9) is an enzyme encoded by the PCSK9 gene on chromosome 1 in humans^10^. PCSK9 was initially discovered as a protein expressed in the brain. Later, it has been described to be mainly expressed in hepatocytes as well as in the kidney, the pancreas, and the small intestine^11^. The three-dimensional (3D) structures of PCSK9 show three distinct domains: the prodomain (aa 31–152), the catalytic domain (aa 153–421), and the C-terminal Cys/His-rich domain (CHRD; aa 453–692), each playing a significant role in managing PCSK9’s biological functions and its trafficking inside cells^12^. The discovery of PCSK9 led to astonishingly fast progress in the understanding of cholesterol homeostasis and the implication of PCSK9 in various pathologies. These include hypercholesterolemia, atherosclerosis, inflammation, sepsis, viral infections, cancer/metastasis, and likely many others. PCSK9 protein acts as a chaperone to escort selected surface receptors toward endosomes/lysosomes for degradation. While the catalytic domain of PCSK9 binds to certain receptors (e.g., LDLR, VLDLR, ApoER2, LRP1), the Cysteine-histidine-rich domain is implicated in the binding of other ones (e.g., MHC class I receptor)^13^.

PCSK9 is involved in the degradation of the low-density lipoprotein receptor (LDLR) and the modulation of intracellular and plasma cholesterol levels, as the binding of PCSK9 to LDLR prevents the removal of LDL particles from the blood plasma^14^.

So, when the plasma PCSK9 levels are elevated because of gain-of-function (GOF) mutations, it leads to the accumulation of plasma LDL-C and an increase in cardiovascular risk. On the other hand, when there is low or no PCSK9 in plasma because of loss-of-function (LOF) mutations, there will be more intact LDLR, which in turn traps more LDL-C from the bloodstream and reduces cardiovascular risk^15^.

In vitro and in vivo studies in both animals and humans have shown that PCSK9, which is strictly linked to hydroxymethylglutaryl- CoA receptors (HMGCoAR) and LDLR pathways, can play a central role in sepsis. Indeed, there is substantial evidence that LDLR participates in the clearance of pathogen's lipids, e.g., lipopolysaccharide (LPS), thus limiting their deleterious pro-inflammatory effect^16^.

Our study aimed to evaluate the prognostic role of serum Proprotein convertase subtilisin/kexin type 9 (PCSK9) level in children with sepsis and severe sepsis.

## Subjects and methods

### Study design and participants

The present study was carried out on 60 children who were admitted to the PICU and in-patient units of Minia University Children's Hospital, El-Minya. They were diagnosed with sepsis and severe sepsis, according to the International Pediatric Sepsis Consensus Conference *(International Pediatric Sepsis Consensus Conference, 2005)* shown in Table 1^17^. Also, 30 apparently healthy children were included as a control group.

**Table 1:** Definitions of systemic inflammatory response syndrome (SIRS), infection, sepsis, severe sepsis, and septic shock as per Goldstein et al.^17^.

SIRS The presence of at least two of the following four criteria, one of which must be abnormal temperature or leukocyte count: Core_a_ temperature of 38.5 °C or 36 °C Tachycardia, defined as a mean heart rate 2 SD above normal for age in the absence of external stimulus, chronic drugs, or painful stimuli; or otherwise unexplained persistent elevation over a 0.5- to 4-h time period OR for children < 1 yr old: bradycardia, defined as a mean heart rate < 10th percentile for age in the absence of external vagal stimulus, -blocker drugs, or congenital heart disease; or otherwise unexplained persistent depression over a 0.5-h time period Mean respiratory rate 2 SD above normal for age or mechanical ventilation for an acute process not related to underlying neuromuscular disease or the receipt of general anesthesia Leukocyte count elevated or depressed for age (not secondary to chemotherapy-induced leukopenia) or 10% immature neutrophils
Infection A suspected or proven (by positive culture, tissue stain, or polymerase chain reaction test) infection caused by any pathogen OR a clinical syndrome associated with a high probability of infection. Evidence of infection includes positive findings on clinical exam, imaging, or laboratory tests (e.g., white blood cells in a normally sterile body fluid, perforated viscus, chest radiograph consistent with pneumonia, petechial or purpuric rash, or purpura fulminans)
Sepsis SIRS in the presence of or as a result of suspected or proven infection
Severe sepsis Sepsis plus one of the following: cardiovascular organ dysfunction OR acute respiratory distress syndrome OR two or more other organ dysfunctions
Septic shock Sepsis and cardiovascular organ dysfunction

^a^Core temperature must be measured by rectal, bladder, oral, or central catheter probe.

The studied children were divided into three groups: *Group 1 (Sepsis group)* included 30 children. *Group 2 (Severe sepsis group):* included 30 children. *Group 3 (control group)*: included 30 apparently healthy children age and sex-matched with the previous two groups. The study was conducted from January 2017 to October 2017.

The study was explained in detail to the parents or legal guardians of the participant children, and written consent was taken from them. The study was designed to respect the expected ethical aspects. It was performed according to the Declaration of Helsinki 1975, as revised in 2008 and approved by the Institutional Review Board and Medical Ethics Committee of Minia University Hospital.

### Baseline clinical assessment

All children included in the study were subjected to complete medical history taken and thorough clinical examination with weight determination. The length of hospital stay for the surviving patients was calculated from the day of admission at the ER department till the day of discharge from the hospital.

We excluded from our study children with sepsis who were older than 16 years, had negative blood cultures, and had normal body temperature or normal heart rate and respiratory rate. Children in the control group were selected from children coming for follow-up in the outpatient clinics after the exclusion of the presence of infection by clinical examination, CBC, and blood culture.

### Laboratory investigations

#### Samples collection

Blood samples were collected from all subjects under complete aseptic conditions for blood culture, hematological, and biochemical laboratory tests. Blood samples were withdrawn in the first two groups at the time of diagnosis of sepsis before initiation of antibiotic therapy.

#### Laboratory methods

Complete blood count (CBC) samples were collected in anti-coagulant EDTA tubes, and CBC was performed immediately by an automated cell counter (Celltac ES, Nihon Kohden Corporation, Automated hematology analyzer, Japan). Arterial blood gases (ABG) were measured by (Sensacore ST-200, India). For cultures, blood was inoculated into blood culture bottles with specific media.

Serum was separated following sample clotting in plain tubes by centrifugation and analyzed immediately for renal, liver, and lipid profiles. They were assayed by an auto chemical analyzer, Konelab 60i (Thermo Electron Incorporation, Finland). Lipids were measured by the fully automated chemistry auto-analyzer system SELECTRA PRO XL (ELI TECH GROUP clinical system) (France). LDL-cholesterol was calculated according to the equation of Friedewald (1972) as follows: LDL-c = TC – {(TG/5) + HDL-c}^18^. The remaining serum was stored at -70°C for further evaluation of PCSK9. Serum PCSK9 was assayed by ELISA (the kit was supplied by Abcam, UK cat. no. ab209884).

### Statistical analysis

Data was coded, entered, and analyzed using SPSS (Statistical Package for Social Sciences) version 26.0. Shapiro–Wilk test was used to determine whether the data followed a normal distribution. Descriptive statistics were calculated and expressed as mean ± standard deviation (SD) or median (Q3-Q1) for quantitative data and as number and percent for qualitative data. T-test was used to compare differences between two independent groups regarding parametric quantitative data and Mann–Whitney test was used to compare two groups regarding non-parametric quantitative data., while the ANOVA and post-hoc test was used to compare quantitative data among more than two groups. The Kruskal–Wallis test was used to determine statistically significant differences between two or more groups of an independent variable on a continuous or ordinal dependent variable. Correlations were performed by using either Pearson’s or Spearman’s correlation coefficient (r). ROC curves analyses were done to determine the sensitivity, specificity and diagnostic accuracy of PCSK9. Linear regression analysis was done to study multiple system dysfunction prediction by PCSK9 increase. The probability of less than 0.05 was used as a cutoff point for all significant tests.

## Results

In this study, the sepsis group included 19 boys and 11 girls, with a mean age of 26.6 ± 32.6 months, ranging from 2 to 120 months. The severe sepsis group included 19 boys and 11 girls, with a mean age of 19.04 ± 28.96 months, ranging from 3 to 96 months. While the control group included 30 apparently healthy children age and sex-matched with the previous two groups; they were 20 boys and ten girls, with a mean age of 32.12 ± 25.44 months, ranging from 2 to 120 months. There were no significant differences between the three studied groups regarding age, sex, and anthropometric measures (p > 0.05). On the other hand, we noted statistically significant differences between them regarding the temperature, heart rate, respiratory rate, platelet count, LFT, and RFT (p < 0.05). On comparing the outcome, there were eight non-survivors in the severe sepsis group, which was statistically significantly more frequent than the other two groups who were all survivors (p < 0.001) (Table 2).

**Table 2:** Demographic, clinical, vital, laboratory data and outcome of the studied groups.

Variable	Group1 Sepsis (n = 30)	Group2 Severe sepsis (n = 30)	Group3 Control (n = 30)	p-value	p1	p2	p3
Age (month): median (Q3–Q1)	10.5 (36–3)	8 (15.8–4.6)	27 (44.3–11.3)	0.08	0.5	0.8	0.2
Sex male: n (%)	19 (63.3%)	19 (63.3%)	20 (66.7%)	0.9	1	0.9	0.9
Weight (kg): median (Q3-Q1)	8.25 (13–4.6)	7.5 (9.9–5.4)	12.6 (15–9.8)	0.01*	0.6	0.09	0.01*
Temperature: median (Q3-Q1)	39 (39.2–38.4)	39 (39.2–38.9)	37 (37.03–37)	0.001*	0.2	0.001*	0.001*
RR							
Tachypnea: n (%)	30 (100%)	7 (23.3%)	0 (0%)	0.001*	0.3	0.002*	0.001*
MV: n (%)	0 (0%)	23 (76.7%)	0 (0%)				
Normal: n (%)	0 (0%)	0 (0%)	30 (100%)				
HR: Tachycardia: n (%)	30 (100%)	30 (100%)	0 (0%)	0.001*	1	0.001*	0.001*
CBC: platelets: (× 10^3^/mm): median (Q3-Q1)	272.5 (310–210.3)	154.5 (210.8–61.8)	310 (326–273.3)	0.001*	0.001*	0.001*	0.001*
LFT							
ALT: (U/L): median (Q3-Q1)	23 (30.8–17.3)	33 (192.3–23.3)	20.5 (25–16.8)	0.001*	0.004*	0.9	0.008*
AST: (U/L): median (Q3-Q1)	23 (29.8–18)	40 (184.3–20.3)	22.5 (27.3–17.8)	0.001*	0.004*	0.9	0.01*
RFT							
Urea: (mmol/L): median (Q3-Q1)	25.5 (35–21)	32 (43.8–23)	24 (26–22.8)	0.02*	0.1	0.6	0.02*
Creatinine: (mg/dl): median (Q3-Q1)	0.6 (0.8–0.4)	0.7 (1.05–0.5)	0.55 (0.63–0.48)	0.01*	0.03*	0.8	0.01*
Survivors: n (%)	30 (100%)	22 (73.3%)	30 (100%)	0.001*	0.001*	0.001*	1

MV: Mechanical ventilation; CBC: complete blood count; LFT: liver function tests; ALT: alanine transferase; AST: aspartate transferase; RFT: renal function tests. p1 = sepsis vs. severe sepsis; p2 = sepsis vs. controls; p3 = severe sepsis vs. control.

*Statistical significance at p-value < 0.05.

Regarding the bacteria identified in blood culture, they were *Staphylococcus aureus* (20%), *Klebsiella* (40%), *Streptococcus pneumoniae* (6.67%), *Escherichia coli* (20%), *Pseudomonas aeruginosa* (6.67%) and *Enterobacter* (6.67%).

We found that LDL-C and PCSK9 were significantly higher in each of the two sepsis groups than the control group, as p ≤ 0.001 in all, except for PCSK9 in the sepsis group when compared to the control group, as p = 0.01. Moreover, LDL-C and PCSK9 were significantly higher in the severe sepsis group when compared to the sepsis group, as p = 0.04 and ≤ 0.001, respectively (Table 3).

**Table 3:** LDL-cholesterol and PCSK9 of the studied groups.

Variable	Group1 Sepsis (n = 30)	Group2 Severe sepsis (n = 30)	Group3 Control (n = 30)	p1	p2	p3	p
PCSK9 (ng/ml): median (Q3-Q1)	164.95 (181.2–144.6)	368.75 (413.6–254.3)	161.85 (176–147.7)	0.01*	0.001*	0.001*	0.001*
LDL-C (mmol/L): mean ± SD	117.5 ± 18.2	131.3 ± 23.2	89.8 ± 23.6	0.001*	0.001*	0.04*	0.001*

p_1_ = control vs sepsis; p_2_ = control vs severe sepsis; p_3_ = sepsis vs severe sepsis, p = among the 3 groups.

PCSK9: Proprotein convertase subtilisin/kexin type 9; LDL-C: low density lipoprotein cholesterol.

* Statistical significance at p < 0.05.

Within the severe sepsis group, non-survivors had significantly higher PCSK9 and LDL-C than survivors (p = 0.01 for both) (Table 4).

**Table 4:** PCSK9 level in children with severe sepsis survivors versus non-survivors.

Variable	Survivors (n = 22)	Non-survivors (n = 8)	p-value
PCSK9 (ng/ml): median (Q3-Q1)	173.2 (220.3–145.4)	341.45 (413.6–254.3)	0.01*
LDL-C (mmol/L): mean ± SD	117.47 ± 18.7	136.45 ± 22.19	0.01*

PCSK9: Proprotein convertase subtilisin/kexin type 9; LDL-C: low density lipoprotein cholesterol.

*Statistical significance at p-value < 0.05.

**Figure 1:**
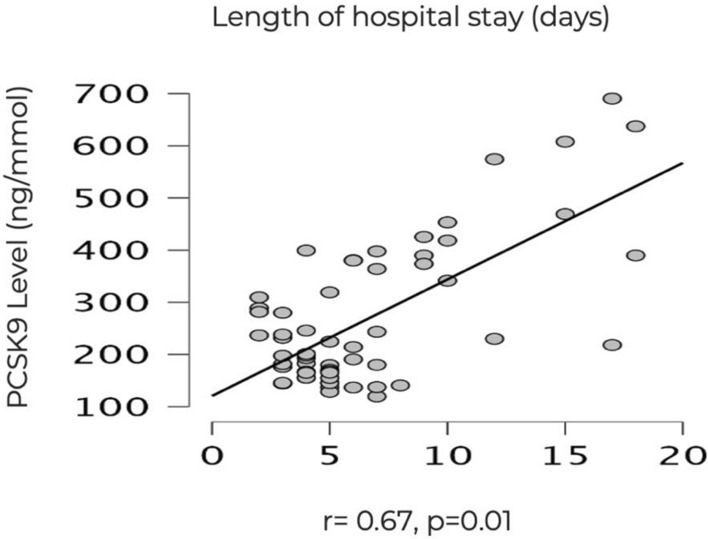
Correlation between PCSK9 level and length of hospital stay.

When Pearson’s and Spearman’s correlations were done to study the associations between length of stay of the surviving children in the two-sepsis groups and PCSK9 and LDL-C levels, we found a significant positive association between length of hospital stay and PCSK9 level (r = 0.67, p = 0.001) (Fig. 1), while the association between length of hospital stay and LDL-C was of statistical insignificance (r = − 0.4, p = 0.8). Moreover, there was no significant correlation between PCSK9 and LDL-C (r = 0.37, p = 0.05).

On comparing the PCSK9 level in the two sepsis groups according to different organ dysfunction developed, children with sepsis or severe sepsis who developed any organ dysfunction had higher PCSK9 when compared to children with sepsis and severe sepsis who did not suffer that organ dysfunction, as p ≤ 0.001 in all, except for renal and hepatic dysfunction, where p = 0.004 and 0.008 respectively.

While linear regression analysis showed that PCSK9 had only predicted hematological dysfunction significantly (adjusted B = − 96.95, p = 0.03) (Table 5).

**Table 5:** Linear regression analysis of multiple system dysfunction predicted by the increase in PCSK9.

Risk factor	Adjusted B	p-value
Cardiovascular	− 38.74	0.4
Respiratory	− 91.88	0.05
Hematological	− 96.95	0.03*
Renal	− 72.58	0.27
Hepatic	− 64.86	0.1

*Statistical significance at p-value < 0.05.

ROC curves analyses of PCSK9 for assessing organ dysfunction showed high sensitivity and diagnostic accuracy are shown in Table 6.

**Table 6:** ROC curves analyses of PCSK9 for assessing organ dysfunction.

System dysfunction assessed	Accuracy (%)	Positive predictive value (%)	Negative predictive value (%)	Cut off-point (ng/ml)	Sensitivity (%)	Specificity (%)
Cardiovascular	84.8	71.4	96.6	> 224.7	95.24	79.49
Respiratory	89.3	80.6	100	> 201	100	82.9
Renal	84.8	20	100	> 281.6	100	71.43
Hematological	82.2	42.3	97.1	> 231.9	91.67	68.7
Hepatic	80.9	35.7	100	> 224.7	100	64

## Discussion

In this study, the sepsis group had significantly higher PCSK9 levels than the control group, which is in agreement with the results of Boyd and his coworkers in 2016^19^. This can be attributed to the fact that in the early hours of sepsis, there is a significant decline in plasma cholesterol levels, which stimulates hepatocytes to increase PCSK9 production^20^. Cholesterol levels may decrease dramatically, secondary to the drop in LDL-C level. The possible mechanisms causing LDL-C drop may be secondary to bacterial endotoxin, pathological lipopolysaccharides (LPS), tumor necrosis factor, interleukin-2, and interferon beta, all potentially present during systemic infection^21^. Furthermore, sepsis-induced LPS have recently been found to have an upregulating effect on sterol regulatory element-binding protein 2, an important transcription factor of the PCSK9 gene^19,22^. PCSK9 binds to LDL receptors (LDLR), preventing clearance of LDL-C via receptors from plasma, which in turn increases serum LDL-C in sepsis^23^. Findings in this study support this, as the sepsis groups had significantly higher LDL-C levels than the control group. Another mechanism was revealed by a recent study on pediatric host response to sepsis. They suggested a potential direct role of the PCSK9-LDLR pathway on vascular homeostasis by its direct effect on angiotensinogen-1 in the developing host with septic shock and that effect may lead to the development of new pediatric-specific sepsis therapies^24^

Although PCSK9 level had a positive association with LDL-C level (r = 0.25), this association was statistically insignificant; this disagreed with Lakoski et al. and Boyd et al.^19,25^. Both studies found a significant positive association between PCSK9 and LDL-C. The insignificance of the correlation in our study may be explained by the additional direct upregulating effect of the pathological LPS on PCSK9 expression, independent from the LDL-C drop stimulatory effect.

The severe sepsis group in this study had significantly higher PCSK9 and LDL-C levels than the sepsis group, as an increase in PCSK9 levels was reported with the development of organ failure in sepsis^26^. PCSK9 hinders LDLR function to preserve LDL-C. It simultaneously hinders the clearance of pathogenic lipids via the LDLR^27^, which in turn leads to organ failure occurring in sepsis^19^ that further aggravates cell damage and systemic inflammation, which directly induces more production of hepatic PCSK9, which leads to further organ failure, and so on^20^. In addition, a recent study in 2022 reported that increased PCSK9 expression during sepsis activates pathways that induce inflammation, which results in vascular endothelial dysfunction and decreased survival rates^28^. This can be confirmed by the fact that children in our study with sepsis or severe sepsis who developed any organ dysfunction had higher PCSK9 when compared to those who did not suffer organ dysfunction.

Non-survivors in this study had significantly higher PCSK9 than survivors, and PCSK9 level was positively correlated with the length of stay in the hospital in the surviving children. Two previous cohort studies supported the association between PCSK9 and sepsis outcomes. They analyzed human genetic data of patients who had been treated for sepsis. It has been demonstrated that subjects with at least one loss of function (LOF) PCSK9 allele were more likely to survive, whereas subjects carrying the gain-of-function (GOF) allele were less likely to survive^26^. Moreover, a study by Shu and his coworkers in 2023 reported that the 28-day mortality of sepsis increased significantly as the baseline circulating PCSK9 level exceeded 370 ng/ml in their patients, indicating circulating PCSK9 levels may be a potential biomarker to predict the prognosis of sepsis^29^.

In our study, PCSK9 was a significant predictor for hematological dysfunction occurring in sepsis. Meanwhile, Boyd and his coworkers found that PCSK9 predicted significant cardiovascular dysfunction^19^. Many studies have studied the association between PCSK9 and many hematological parameters, and a possible link between hematological changes and increased cardiovascular risk was suggested. Li et al. reported an association of plasma PCSK9 levels with white blood cells and their subsets^30^, and recently, an inflammatory environment was reported to induce the generation of PCSK9 in macrophages. In addition, the presence of PCSK9 would directly trigger the inflammation mediated by monocytes and macrophages^31^. Another study reported that the ABO group might be a significant determinant of plasma PCSK9 level and coronary artery disease (CAD) susceptibility^32^. A third study by the same study group reported a potential link between PCSK9 and platelet count in patients with coronary artery disease. They suggested that this link may be involved in atherosclerosis and metabolic disorders^33^. Adding to the previous studies, a study in 2017 by Gurbel et al. demonstrated the possible influence of PCSK9 on inflammation and platelet reactivity^34^.

## Conclusion

PCSK9 represents an innate immune system response to sepsis. Its release is aggravated in sepsis complicated with organ dysfunction. Also, its level was higher in children who did not survive and was positively correlated to the length of hospital stay in the surviving children. So, PCSK9 can be used as a prognostic biomarker for early prediction of organ dysfunction and mortality risk in pediatric sepsis syndrome.

## Study limitations

Further multicenter studies with a larger number of patients are needed to confirm its suitability in clinical practice and its relation to other sepsis morbid outcomes.

## Data Availability

The datasets used and analyzed during the current study are available from the corresponding author upon reasonable request.

## Abbreviations

ABG: Arterial blood gases;
CBC: Complete blood count
GOF: Gain-of-function
HDL-cholesterol: High-density lipoprotein-cholesterol
HMGCoAR: Hydroxymethylglutaryl-CoA receptors
LDL: Low density lipoprotein
LDL-C: Low density lipoprotein-cholesterol
LDLR: Low density lipoprotein receptor
LOF: Loss-of-function
LPS: Lipopolysaccharide
LFT: Liver function tests
RFT: Renal function tests
PCSK9: Proprotein convertase subtilisin/kexin type 9
